# Revisiting the hospital-issued gown in hospitalizations from a locus of control and patient-centered care perspectives: a call for design thinking

**DOI:** 10.3389/fpubh.2024.1420919

**Published:** 2024-09-16

**Authors:** Gillie Gabay, Hana Ornoy

**Affiliations:** ^1^Sciences, Achva Academic College, Arugot, Israel; ^2^Business School, Ono Academic College, Kiryat Ono, Tel Aviv District, Israel

**Keywords:** design thinking, hospitalization, patient-engagement, patient gown, patient-centered care, locus of control, patient-clinician communication

## Abstract

**Introduction:**

Patient-centered care (PCC) is the preferred health policy approach that emphasizes responding to individual patient preferences, wishes, and needs. PCC requires active patient engagement. While there has been extensive research on physicians' robes, there is limited research on hospital-issued patient gowns during hospitalizations. How does the gown affect the cognitive–emotional experience of hospitalized patients? How is the gown associated with PCC?

**Methods:**

The sample of this cross-sectional study consisted of 965 patients who were hospitalized at least once during the past year in a tertiary hospital. Measures were previously published.

**Results:**

The gown was strongly associated with lack of control and increased distress, and was negatively associated with patient proactiveness, engagement, and taking responsibility for self-management of chronic illness. Compared to male patients, female patients wearing the gown had stronger negative emotions and cognitively strong associations with the external locus of control, which inhibited engagement.

**Discussion:**

The hospital gown is an unacknowledged barrier to achieving PCC, inhibits patient engagement, and reflects the paradoxes of inadvertently excluding patients' needs from hospital practice. The hospital gown must be modified to protect the patient's voice and enhance engagement. Policymakers are called to apply design thinking to facilitate patient participation in decision-making to accord hospital clothing to PCC and improve healthcare delivery.

## 1 Introduction

Patient-centered care (PCC) is the preferred policy for delivery of care (1–6). PCC is care that fundamentally responds to individual patient preferences, wishes, needs, and values, ensuring that patient values direct decisions (7). This policy was proven to reduce costs while improving clinical outcomes, patient experiences, and provider experiences (1–4). Delivery of the PCC policy stresses the patient–provider relationship and aims at optimizing the use of information that patients provide to achieve the outcomes that are most important to patients (1–8). As health systems move toward value-based care, PCC becomes foundational to delivering high-quality care, especially because it is fully consistent with population health management, which improves the health of the whole population (9, 10). Therefore, implementing PCC is a high priority for hospitals, but hospitals are far from achieving PCC (11, 12). Acknowledged barriers to achieving PCC are lack of knowledge on integrating PCC into practice, deficient skills of physicians, and professional burnout (13–15). This study focuses on the patient gown within PCC as an additional barrier to PCC, calling for design thinking to remove this barrier.

The PCC policy intentionally directs patients to play a central and active role as meaningfully engaged and responsible for their health (16, 17). Patients move along a continuum from passive to active engagement (18–22). An acceptable definition of patient engagement focuses on the relationship between patients and providers as they work together, during and beyond the care experience, to promote and support active patient involvement in healthcare, strengthening their influence on decisions, at both the individual and collective levels (23–25). To be engaged, patients need to become active in obtaining knowledge and acquiring skills and confidence for managing their illness (26). Patient engagement can occur in direct care, organizational design, and policymaking, influencing patients' capacity to be engaged, leading to improved quality of care, better outcomes, and greater cost efficiency (23). Patient engagement ranges along a continuum characterized by the amount of information that flows between patient and provider, the extent to which the patient adopts an active role in decisions, and the extent to which patient organizations influence decisions of healthcare organizations and policies cultivating engagement (23). Thus, patient engagement refers to the cognitive and emotional conditions that patients express through observable behaviors of the authentic self (27).

At the patient–provider level, every interaction may foster patient engagement that, in turn, can facilitate patient trust, resulting in higher medication adherence, fewer readmissions, and self-management of illness (22, 28). Many stakeholders are therefore interested in patient engagement in capturing the patient's voice and incorporating it into practice (29, 30). PCC differs from patient engagement, conveying a vision of healthcare as a partnership between providers and patients, who become active, informed, and influential, to ensure that decisions respect the preferences, needs, and beliefs of patients (31, 32).

Physicians are encouraged to facilitate PCC when interacting with patients by helping them become knowledgeable regarding their illnesses, express their expectations, and share decision-making (33–35). Physicians, however, identified patient reluctance to be engaged as a barrier to achieving PCC (33, 34). PCC encourages patients to ask questions, share their anxiety, take on an assertive approach to reciprocal communication, and share their health-related concerns when interacting with physicians (15, 36, 37). Communication of patients, however, may be affected by the hospital-issued gown, which is an integral component of the hospital environment across countries (30, 38, 39).

Hospital gowns are routinely worn by hospitalized patients as a form of standardized attire in hospitals in the U.S., Canada, China, the Middle East, Italy, Hungary, and the UK (40–43). The hospital gown provides benefits to all stakeholders (44–46). For patients, the gown provides an accessible clothing option during their hospitalization that can be easily changed in the event of incontinence, bleeding, etc. Gowns are meant to be functional, non-constricting, and provide patients with an effortless experience (47). For clinicians, the gown allows easy care for patients who are sedated, unconscious, in surgical or intensive care settings, weak, or with mobility difficulties (47). For hospitals, gowns are an easy, one-size-fits-all, cheap, versatile, easily washed, and re-used attire (47). In the past decade, tremendous efforts were invested in developing tools to assess patient engagement and indicators of PCC, but insights from the patients, as primary stakeholders, regarding the hospital gown and its effect on their capacity for engagement are insufficient (1, 9, 48–50).

The gown is a symbol of the passage into a new role as a patient, carrying low status due to worn-out clothing, lacking privacy, and constructing the social identity of hospitalized patients (38, 51–54). A handful of qualitative studies found that the hospital gown can be perceived as dehumanizing, as the gown was associated with lower wellbeing of inpatients due to an increased sense of vulnerability, feeling more exposed, self-conscious, uncomfortable, cold, embarrassed, disempowered, and because the gown limited patients from being active (55–59).

Thus, although PCC stipulates a two-sided, mutually involved communication and calls for patients to be engaged, the patient's hospital gown may affect cognition and emotions, inhibit self-expression, and negatively impact patient engagement when interacting with physicians. Research on the cognitive–emotional effect of the gown on patient engagement within the PCC framework is scant (60). The importance of patient engagement when wearing the hospital gown calls for revisiting the issue of patient attire during hospitalizations. Self-expression while wearing the gown may also be affected by the locus of control (LOC).

LOC is the most frequently studied perception in healthcare (58). The social learning theory classifies people along a continuum of perceived control ranging from internal locus of control (ILOC) to external locus of control (ELOC) (59). People with an ILOC believe that success or failure is due to their own efforts and consequently take self-directed actions, while people with an ELOC believe that their achievements are directed by luck, chance, or others (60). Traditionally, ILOC reflected personal mastery and referred to the assumed internal states of individuals who seek information, have higher alertness, and make decisions (59). People with ILOC actively and willingly rely on internal resources to deal with difficult circumstances (61).

A study on patients' trust in physicians extended the concept of ILOC beyond personal attributes to one's perceived ability to resolve health-related problems and to interact more effectively with physicians (62). While patients with ILOC were proactive and took responsibility for improving their health, patients with ELOC believed that they could influence their health or recovery, and they depended on chance, externality, and physicians. Patients with high ILOC were more active in interactions with physicians and used efficient problem-focused coping strategies (55). Hospitalized patients with ILOC were able to make more effective decisions, had higher self-efficacy, and took care of themselves (63). A significant positive relationship was found between ILOC and high adjustment to situations linking ILOC to better healing (64–67). The association between the gown and LOC is understudied (39, 55). What is the association between wearing the hospital gown and the LOC of patients? Is the gown a potential barrier to achieving PCC?

Patients' sense of self is defined through interactions with others (68). Patients may adjust their engagement behaviors with physicians, based on perceived role relations, relative power, and status (69). The patient's inner world underlies interactions either orienting the patient toward engagement or away from it in interactions with physicians (70). We explored the cognitive–emotional effect of the gown on patients and its association with their capacity to be engaged when wearing the hospital gown. Much has been written about the symbolic function of the whitecoat, but the patient gown has not received similar attention (39). From a PCC perspective, it is essential to understand the association of the gown with the patient's emotions and LOC, which may shape self-expression and patient engagement during hospitalizations. The research question was “how is the patient gown in hospitalizations associated with the emotions and the LOC of patients?”

Since the patient's gown is a symbolic embodiment of the “sick” role, namely, relinquishing control to clinicians and experiencing emotional vulnerability (71, 72), we expected that the emotions of patients when wearing the gown might be related to LOC forming an inclination to proactively communicate with physicians during hospitalizations. A previous study found that female patients felt more exposed, self-conscious, vulnerable, uncomfortable, cold, embarrassed, and disempowered when wearing the gown compared to male patients and patients with a short illness (73). We, therefore, expected that the negative emotions of patients when wearing the gown would be related to higher ELOC in chronically ill patients and female patients. Furthermore, we expected that compared to male patients, female patients might have stronger negative emotions when wearing the gown and stronger associations with ELOC. Finally, we expected that compared to male patients with a short illness, chronically ill female patients would have stronger negative emotions when wearing the gown, which would be associated with ELOC.

The few studies that were performed on the patient gown were qualitative. This is a quantitative study testing the association between the gown and the LOC of hospitalized patients.

## 2 Methods

### 2.1 Ethical approval and sample size

The ethics board of the academic institution with which the first author is affiliated granted ethical approval (IRB# 117). “G^*^Power (v. 3.1.9.7) statistical software was utilized to determine the minimal sample size for the analyses using a standard α error probability of 5%, a power of 95%, and a fixed effect size of 0.15 for a ratio of 5:1 (predictors: outcomes); the *minimal a-priori* sample size is *n* = 138 (and *n* = 204 for an effect size of 0.10). Based on these *a-priori* analyses, a sample size above 204 (as the stricter upper bound) is considered adequate for the subsequent analysis. The sample consisted of 1,008 Israeli participants who were hospitalized in the 6 months in tertiary public hospitals.

### 2.2 Procedure

Following ethical approval, this cross-sectional research was carried out through the academic research laboratory using a digital questionnaire. A digital questionnaire was created. Students pursuing a bachelor's degree in health management, who have studied quantitative and qualitative methods, identified a relative or acquaintance who was hospitalized in the last 6 months. Each student screened the participant for the department in which she or he was hospitalized, omitting new mothers from maternity wards and people who were hospitalized and discharged from the emergency department.

Students explained the study goals, and once participants agreed, students sent them the digital link to the questionnaire. The purpose of the research was to learn about the inpatient experience when wearing a gown. Participants marked a box in the introduction part of the questionnaire for their consent for participation and publication. Older participants who were not averse to technology had the questionnaire read to them. The average time to fill out the questionnaire was 10 min. From July 2022 to July 2023, 1,008 questionnaires were completed under the supervision of the second author.”

### 2.3 Measures

To assess *Patients' feelings when wearing a hospital gown*, the Cogan et al. (44), a 34-item questionnaire was used. An example item is “I feel vulnerable when wearing the gown” (α = 0.67).

*To assess Perception of the gown*, the Frankel et al. (74) six-item questionnaire was used. An example item was “I was able to move about easily when walking and while in bed” (α = 0.92).

*To assess the locus of control*, the 10-item brief questionnaire was used (75). An example item is: “My quality of life is affected by others” (α = 0.70).

*Demographic control variables* were age, gender, education, family status, religious affiliation, religiousness, ethnicity, number of children, occupational status, and household income.

*Health control variables* were hospitalization frequency, type of illness, and department in which the patient was hospitalized.

## 3 Results

### 3.1 Descriptive statistics

After deleting missing data, this study included 956 inpatients, with a mean age of 26.5 years (SD: 7.01), ranging from 16 to 67 years, of which 56.5% were women and 46.5% were men. Most of the patients reported being single (57%), and 40.8% stated that they were married or in a relationship. The average completed school years was 10.3 (SD: 5.7), ranging from 8 complete school years to 21 years. The majority of the patients were identified as Jewish (96.2), and the rest were either Muslims or Christians (3.1% and 0.8%, respectively).

Most patients self-identified as traditional (26.3%), followed by secular (26.1%), religious (20.7%), partially traditional (14.9%), and very religious (12.1%). Most patients reported an average household income (62.6%), 23.7% reported a higher-than-average income, and 13.7% reported a lower-than-average income.

The distribution of the hospitalization frequency was as follows: (a) 2.4% were hospitalized once in half a year; (b) 0.6% once in 3 months; (c) 0.9% once in 1–2 months; (d) 6.1% once a year; (e) 23% once every 5 years; and (f) 66.9% once every 10 years. Most of the respondents (90.2%) did not have any prolonged or chronic disease, while only 9.8% reported they did. Approximately 10% of the patients reported being hospitalized once a year or more, and 89.9% reported a hospitalization once every 5 years or less. ELOC average was 3.42 (SD: 0.48), ranging from 1.8 to 4.9. Perceptions of the gown average was 2.7 (SD 0.64), ranging from 1 to 5. Table 1 presents the sample demographic distribution.

**Table 1:** Sample demographic distribution.

**Variable**	**Mean (SD)**	**Range**	**Distribution**
Age	26.5 (7.01)	16 to 67	Female patients (56.5%) and male patients (43.5%).
Family status			Single (57%), and married/in a relationship (40.8).
Education	10.3 (SD 5.7)	8-21	
Religion			Jewish (96.2) and Muslims or Christians (3.1% and 0.8%, respectively).
Religiosity level			Traditional (26.3%); secular (26.1%), religious (20.7%), partially traditional (14.9%), and very religious (12.1%).
Income Level			Average household income (62.6%), higher-than-average income (23.7%), and lower-than-average income (13.7%).
Hospitalization frequency			(a) 2.4% were hospitalized once in half a year, (b) 0.6% once in 3 months, (c) 0.9% once in 1–2 months, (d) 6.1% once a year, (e) 23% once every 5 years, and (f) 66.9% once every 10 years.
Chronic disease			9.8%
Hospital frequency:			Once a year or more (10%) and once every 5 years or less (89.9%)
External control	3.42 (SD: 0.48)	1.8 to 4.9	

### 3.2 Pearson correlation tests and independent T-tests

The Pearson correlation tests were performed to test the relationships between negative emotions when wearing the gown and ELOC. Negative significant correlations were found between each emotion and ELOC including aggravation (r = −0.16, *p* < 0.001), alienation (r= −0.16, *p* < 0.001.16), sadness (r = −0.15, *p* < 0.001), and victimization (r = −0.16, *p* < 0.001). Table 2 presents the emotional central tendencies and their distributions.

**Table 2:** Emotional central tendencies and distributions.

	**Mode**	**Median**	**Mean**	**SD**	**Minimum**	**Maximum**
Aggravation	3	3.2	3.15	0.87	1	5
Blackout	3	3	2.77	1.02	1	5
Sadness	3	3	3.04	1.12	1	5
Victimized	3	3	2.9	0.88	1	5

Independent T-tests were performed to compare male patients and female patients on their perceptions of the gown, negative feelings, and ELOC. Significant differences were found between male patients and female patients. Compared to male participants, the mean score of perceptions of the gown was lower in female participants, as was the mean score of ELOC, while the mean score of all negative emotions was higher for female participants. Table 3 presents the t-test results.

**Table 3:** Independent *t*-tests comparing male participants and female participants.

	***T***	**Df**	***P***	**Mean**	**SD**
Perception of the gown	5.94	891	< 0.001	M:2.84 F:2.59	M:0.6 F:0.66
ELOC	4.09	893	< 0.001	M:3.49 F:3.36	M:0.51 F:0.45
Aggravation	4.35	785	< 0.001	M:3 F:3.27	M:0.86 F:0.87
Blackout	3.64	797	< 0.001	M:2.63 F:2.88	M:0.98 F:1.03
Sadness	5.4	809	< 0.001	M:2.8 F:3.22	M:1.07 F:1.13
Victimized	4.82	784	< 0.001	M:2.72 F:3.03	M:0.86 F:0.88

Next, the perceptions of the gown. The positive perceptions of the gown were rated low to medium on a scale from 1 to 5. “I am able to move better when wearing the gown” (M = 1.95, SD = 0.98); “It is easy to wear the gown” (M = 2.14, SD = 1.12); “I feel protected when wearing the gown” (M = 2.48, SD = 1.25); I feel comfortable when wearing the gown” (M = 2.50, SD = 1.27). Negative emotions when wearing the gown and ELOC were compared among male participants and female participants, and significant differences were found. Perceptions of the gown and ELOC were higher in male participants, while all negative emotions were higher in female participants. Finally, participants with chronic illnesses were compared with participants with short-term illnesses. In participants with a chronic illness, ELOC was not significantly different. The perceptions of the gown were significantly higher in male participants, yet all negative emotions were significantly higher in female participants, as presented in Table 4 by gender and type of illness.

**Table 4:** Independent t-tests comparing perceptions of the gown, negative emotions, and ELOC by gender and type of illness.

	***T***	**Df**	***P***	**Mean**	**SD**
**By gender**					
Perception of the gown	2.59	87	0.006	M: 2.87 F:2.52	M: 0.65 F:0.61
ELOC	0.453	84	Ns	M: 3.51 F: 3.46	M: 0.47 F: 0.48
Aggravation	3.3	72	< 0.001	M: 2.76 F: 3.42	M: 0.92 F: 0.78
Blackout	2.46	74	0.008	M: 2.43 F: 2.94	M: 0.81 F: 0.92
Sadness	3.24	76	< 0.001	M: 2.56 F: 3.35	M: 1.08 F: 1.05
Victimized	1.83	74	0.035	M: 2.68 F: 3.02	M: 0.86 F: 0.75
**By type of illness**					
	5.38	775	< 0.001	M: 2.84 F: 2.6	M: 0.59 F: 0.66
ELOC	4.15	776	< 0.001	M: 3.5 F: 3.35	M: 0.51 F: 0.44
Aggravation	3.53	687	< 0.001	M: 3.03 F: 3.26	M: 0.86 F: 0.87
Blackout	3.07	695	0.001	M: 2.64 F: 2.88	M: 1.01 F: 1.03
Sadness	4.45	706	< 0.001	M: 2.83 F: 3.2	M: 1.07 F: 1.13
Victimized	4.35	684	< 0.001	M: 2.73 F: 3.03	M: 0.87 F: 0.89

## 4 Discussion

Failing to meet patient preferences to be engaged was significantly associated with dissatisfaction, distrust, and reduced capacity for self-management of illness post-discharge (29, 76). The gown was associated with a lack of control and increased distress and was negatively associated with ILOC, which comprised proactiveness, learning, and taking responsibility (62). The findings support a previous study in which the patient gown caused psychological distress, disempowerment, and lower self-esteem, as well as deepened the intense vulnerability of hospitalized patients (77, 78). The self-concept of the patient was lacking control when adhering to the standard of wearing a gown upon hospitalization, thereby impeding patient engagement and PCC achievement.

Our findings suggested that the gown failed to meet the needs of patients and was negatively associated with ILOC, which was essential for active patient engagement, effective communication with clinicians, and disease management (17–21, 62, 79).

The gown was associated with sadness, alienation, and victimization, all of which were inconsistent with PCC (80, 81). Looking at the patient's inner world as underlying communication with physicians, the cognitive–emotional experience of wearing the gown may diverge patients from engaging with physicians (82). The findings indicated that the patient's experience with the gown was related to ELOC, inhibiting engagement (62, 64, 67). The gown seems to be paramount to a patient's dignity in hospitalizations and may shape an overall negative hospital experience (46). Negative hospital experiences of patients due to hospital culture, work environment, and providers' deficient capacity to provide PCC contributed to provider dissatisfaction and burnout (83). Therefore, addressing patient experiences may have the double benefit of improving patient care and reducing provider burnout.

Although achieving PCC is a primary goal, patient needs and emotions are not incorporated into the co-design of clothing in hospitalizations, which is solely controlled by policymakers and hospitals. Thus, while the PCC policy and medical practice advocate PCC calling for respect, privacy, and dignity as fundamental cornerstones in the care of each individual patient, when patients remove their own clothes and wear hospital gowns, their self-expression as individuals is diminished (3). In addition to the physical vulnerability that requires hospitalization, the negative emotional effect of the gown on LOC is an additional barrier to PCC, strengthening ELOC and weakening the patient as an engaged partner in the communication rather than empowering the patient as an engaged partner.

The acceptance of the backless hospital gown when advocating PCC is a clash that should challenge current policies and practices and encourage new moral thinking and new policies relating to hospital clothing to improve the emotional experience of hospitalized patients and promote engagement and PCC. Policymakers and hospital management must evaluate the messages emerging from the requirement to wear the gown vis-à-vis the importance of implementing PCC as a primary goal (70). The patient gown may represent the lower end of the engagement continuum, where the flow of information is in one direction, and patients have limited power for decision-making. At the lower end, organizations and policymakers define their own agendas, and information flows bi-directionally and then back to the system (23).

**Figure 1:**
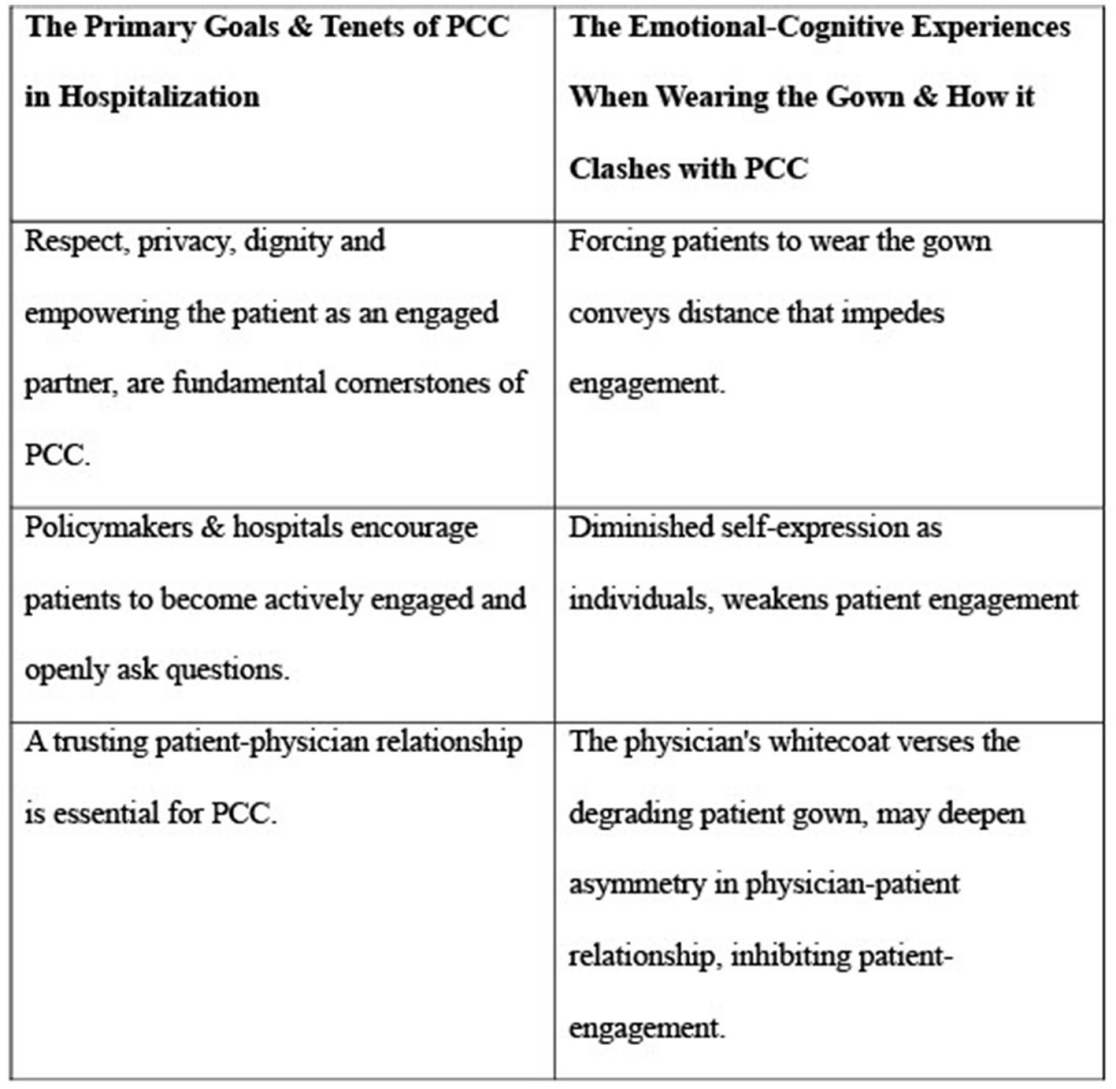
Misalignment of the hospital-issued patient gown with patient-centered care. Clashes between the primary goals and Tenets of PCC in Hospitalization and the emotional-cognitive experiences when wearing gowns.

A second clash is between the declarations of hospitals that patients should openly ask questions and the policy of forcing patients to wear gowns. This gown requirement conveys inequality and distance, diminishing individuality, impeding engagement, and inhibiting the primary goal of PCC. A third clash relates to the patient–physician relationship, which is essential for PCC (8). This relationship has been critically viewed as asymmetrical in power, inhibiting patient engagement (84). Physicians have the knowledge, enable treatment, possess control over how decisions and what decisions are made, and may use a range of strategies to shape interactions with patients (85). The whitecoat of the physician vs. the degrading gown of the patient may manifest an additional asymmetry that, although common, raises ethical concerns about how the patients are treated as engaged partners. Patients acknowledge the gowns as a “necessary evil”, but it is a medical myth persisting through tradition and serving as a mechanism for anonymity rather than for PCC promotion (55, 86). Figure 1 presents the three clashes between PCC and the patient gown.

While backless gowns, held together with ties at the back, are needed for initial pre-operative and post-operative care of the patient, in the recuperation phase of the hospitalization, the gown impedes PCC. The above three clashes between the primary goal commonly framed to meet patients' needs and the secondary goal of operative care fail to guard against medical paternalism (87, 88). Indeed, there may be tension in hospitals between operative care and PCC delivery, but high-performing hospitals are expected to exhibit an organizational culture of patient empowerment, meeting patient expectations and fostering multidirectional communication that facilitates engagement and PCC (6).

While previous research explained deficient PCC by lack of skills and called for better training for physicians, it is time to raise more fundamental questions regarding the values that are encompassed in the PCC concept (e.g., patient autonomy, engagement, and perceived control) (9, 21). The insights of this study call upon policymakers and hospital managements to not only declare their aspiration to achieve PCC but also to manage the messages that are incorporated in the requirement to wear the gown, consider its meaning within the structure of power, and effectively manage the primary goal of PCC achievement.

Policies that inadvertently exclude patient emotional needs from core practices must be modified to protect the patient's voice, enhance patient engagement, and promote PCC (89). Since the inclusion of the patient voice falls short on hospitalizations in general and specifically, on the emotional–cognitive experience of patients when wearing the gown, policymakers are called upon to redesign the clothing of inpatients. In fact, it is long overdue to challenge the status quo of the open-backed gown, focus on the emotional needs of hospitalized patients, and enhance patient engagement as a partner. The gown should be limited to operative medical necessities, enabling patients to change into clothing they prefer, as soon as possible, to preserve their self-worth and ILOC (29, 90).

To challenge the status quo of the gown requirement and align patient hospitalization clothing with PCC, policymakers are called upon to apply design thinking by conducting meetings to assess what patients, clinicians, healthcare leaders, and policymakers perceive as attire that promotes ILOC and patient engagement and may better translate PCC into healthcare practice (91). Design thinking is a human-centered methodology proposed as a systematic approach to innovation in healthcare through the active participation of patients (92). Design thinking prioritizes patient desires and needs, which implements PCC, resulting in a better understanding of the problem and developing effective solutions (91). Using design thinking to implement an effective change in hospital clothing requires patient participation in the process, not only for input but also as an equitable participant in the decision-making processes on clothing and related work processes (92).

Co-design of the gown may promote engagement at the end of the continuum of high engagement. Patients may be more active partners. Information may flow bi-directionally; patients may communicate with clinicians about their health situation, understand the risks and benefits of treatment options, ask questions, share beliefs and preferences, and decision-making responsibility may be shared, as called for by the PCC strategy (23). At the policymaking level, engagement may focus on developing, implementing, and evaluating national, state, and local healthcare policies. Patients may engage in shaping policies to ensure that they are responsive to the perspectives and needs of patients (23). More active patients may influence clinicians in giving patients timely, complete, and understandable information; elicit patients' values, beliefs, and risk tolerance regarding care choices, and encourage patients to be engaged according to the patient's wishes (93). Patients can propose novel ideas that will create value for all stakeholders, promote engagement, and identify a range of priorities to better achieve PCC in hospitalizations (89).

This novel study is not without limitations. Cultural attributes of inpatients and clinicians, the convenient rather than non-representative sample of hospitalized patients, and the demographic composition of the sample, with only 3.1% Muslims who comprise 22% of the population, and only 0.8% Christians who comprise 4% of the population, limit the generalization of the study. Future studies may replicate this study in diverse populations of hospitalized patients with a larger sample and also test potential differences in experiences for patients wearing a cotton (reusable) gown vs. a polypropylene (disposable) hospital gown.

## Data Availability

The original contributions presented in the study are included in the article/supplementary material, further inquiries can be directed to the corresponding author.
